# Single atom catalysis poised to transition from an academic curiosity to an industrially relevant technology

**DOI:** 10.1038/s41467-021-21152-0

**Published:** 2021-02-09

**Authors:** Abhaya K. Datye, Hua Guo

**Affiliations:** 1 Department of Chemical & Biological Engineering, University of New Mexico, Albuquerque, NM USA; 2 Department of Chemistry and Chemical Biology, University of New Mexico, Albuquerque, NM USA

**Keywords:** Catalyst synthesis, Heterogeneous catalysis, Chemical engineering

## Abstract

During the past decade, initial skepticism rapidly changed into widespread recognition of the role of single atoms in heterogeneous catalysts. The next decade could usher in the era of industrial applications as manufacturing of durable single atom catalysts is perfected.

## The journey from nanoparticles to single atoms

Boudart^1^ defined the concept of turnover rate, also called turnover frequency (TOF)—molecules converted per surface site per second, which allowed the development of benchmarks for catalyst performance that could be reproduced across laboratories. Essential to defining TOF was the measurement of dispersion, namely the fraction of atoms on the surface of a catalytic nanoparticle, typically measured by chemisorption. The ultimate goal of catalyst synthesis was therefore to achieve 100% dispersion, which would allow the highest reactivity to be achieved per atom of the catalyst. The logical next step, “Can a single atom serve as a catalyst?”, was a rhetorical question posed by Sir John Thomas^2^. Any lingering doubts about whether single-atom species on oxide supports could serve as catalysts were dispelled by Qiao et al.^3^, when they demonstrated remarkable CO oxidation activity on catalysts that contained Pt atomically dispersed on FeO_x_.

Isolated single atoms of noble metals were long suspected to be present on supported metal catalysts, but they were largely invisible via high-resolution transmission electron microscopy due to limited resolution and contrast. Imaging of single atoms had been demonstrated by Crewe in the 70s using the technique of high-angle annular dark-field imaging^4^. The atomic number contrast of uranium atoms on a low atomic number carbon substrate made imaging of single atoms possible^4^. Application to supported catalysts started appearing in the late 80s, an early example being the work of Treacy at Exxon^5^, who reported imaging single atoms of Pt in a zeolite. Routine imaging of single atoms in heterogeneous catalysts had to wait for the development of spherical aberration correctors and faster computers that made the alignment of the microscopes possible. Advances in aberration corrected scanning transmission electron microscopy (AC-STEM), with currently available resolution at the sub-Å level, have made possible the identification of individual atoms based on their contrast. Widespread availability of this technique has played a major role in bringing single-atom catalysts to the forefront of heterogeneous catalysis.

**Fig. 1:**
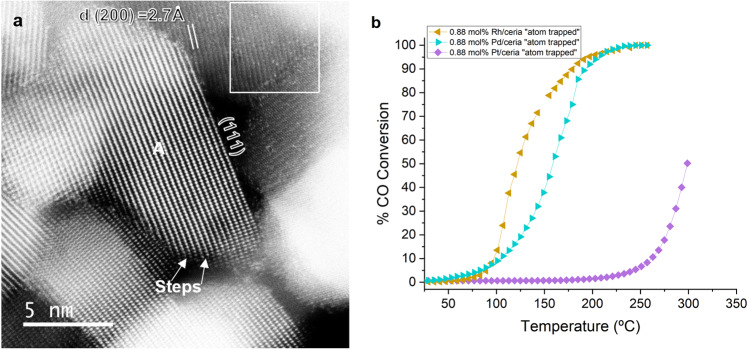
Characterization of single-atom catalysts. **a** AC-STEM image of 3 wt% Pt/ceria prepared via atom trapping. Particle A is oriented along the [112] zone axis resulting in strong contrast from the Ce atom columns, while the ceria particle in the boxed region is oriented off the zone axis making it easier to detect the Pt single atoms, which appear as bright dots. The boxed region is 5 nm × 5 nm in size and contains 25 Pt atoms, consistent with the overall loading of 1 Pt atom/nm^2^. Surface steps are a common feature of ceria polyhedral particles and are likely sites for strong binding of Pt single atoms. The figure content is adapted with permission from Kunwar et al.^6^. Copyright (2019) American Chemical Society. **b** CO oxidation reactivity of Pt, Pd, and Rh single-atom catalysts prepared via atom trapping. The catalysts contain identical molar loading equivalent to 1 wt% Pt/CeO_2_ (0.88 mol% of Pt, Pd, or Rh and 99.12 mol% CeO_2_). The CO oxidation was performed using 20 mg of catalyst, with 1 ml/min of CO, 1.5 ml/min of O_2_, and 75 ml/min of helium. The samples were heated to 300 °C at 2 °C/min and cooled back down to room temperature in the reaction mixture to test for reproducibility. The data shown represent the third run. The catalysts are stable and each of them contain exclusively atomically dispersed metal species located on the surface of the ceria. The figure content is adapted with permission from Alcala et al.^12^. Copyright (2021) Elsevier, Inc.

AC-STEM is unique in that single atoms fade in contrast and go out of focus in the image unless they are located in the focal plane. We can think of the AC-STEM image as a slice through the specimen, even though the image is a projection of a three-dimensional sample. This limited depth of focus makes it possible to image individual single atoms and to even count their number per unit area. AC-STEM is therefore a quantitative technique for single-atom imaging, as shown in Fig. 1a, where the surface concentration of 1 atom of Pt/nm^2^ was inferred by counting 25 bright dots in the 5 nm × 5 nm box^6^. This surface concentration agrees with the calculated value assuming all the Pt in this sample is distributed uniformly over the available surface area of the ceria support. Direct measurements via low-energy ion scattering confirm that the sample contains exclusively single atoms, a distinguishing feature of the method of atom trapping was used by Kunwar et al.^6^ to demonstrate high metal loadings (3 wt%) of isolated Pt atoms on a commercially available ceria support.

## Achieving thermal stability and high reactivity

Single atoms are prone to agglomerate into clusters or nanoparticles through the process of Ostwald ripening. For industrial applications, single-atom catalysts need to be stable under reaction conditions and during accelerated aging. The most widely used catalyst supports are based on oxides, hence there have been intensive studies of binding sites for single atoms^7^. On high surface area alumina, Kwak et al.^8^ proposed that penta-coordinated Al^3+^ sites serve to anchor Pt single atoms. However, the binding to the alumina support is not strong enough to prevent the transformation of single atoms into nanoparticles. Reducible oxide supports such as ceria are capable of stabilizing single atoms by forming covalent bonds, where surface oxygen atoms are shared with the ionic Pt species^9^. The binding energy of ionic Pt species on ceria can exceed that of Pt atoms on bulk Pt metal^10^. This makes it possible to prepare single-atom catalysts by simply heating in air at 800 °C a Pt precursor deposited on a catalyst support (atom trapping)^11^. Surprisingly, atom trapping works very well not only with metals with volatile oxides, like PtO_2_, but also with those that have a very low vapor pressure, such as PdO^12^. Despite identical metal loading, the CO oxidation reactivity of atomically dispersed Pt, Pd, and Rh shows dramatic variations (Fig. 1b), a satisfactory explanation for this variation is still lacking.

The stability and reactivity of single-atom species on support surfaces is ultimately determined by their electronic structure, which can be significantly tuned by the binding environment. To this end, DFT is ideally suited to provide insights, thanks to its high-computational efficiency and ability to characterize transition metals. These calculations confirm that stability and reactivity are strongly influenced by the binding environment, mainly through charge transfer. As a result, the catalytic mechanism is often drastically different from that on metallic nanoparticles. Indeed, the oxidation state of the single metal species may even change during the catalysis^13^, controlled by the nature of the binding species. This is particularly significant for reducible oxides, such as ceria. In this sense, single-atom catalysis is not due to just the single metal atom, but also its surroundings, which can be likened to the metal–ligand interaction in homogeneous catalysis. Single-atom species can also serve as promoters, which are not directly involved in catalysis. In a recent example^14^, atomic Ni dopants were shown to promote the oxygen vacancy on ceria surfaces, which allows the exposure of Ce^4+^/Ce^3+^ species to form a frustrated Lewis acid–base pair in the catalysis of alkyne hydrogenation.

## A technology ready for industrial deployment

The most widely studied reaction on single-atom catalysts is CO oxidation, so we might ask how close are we to commercial applications of single-atom catalysts in emission control systems? In the case of Pt, the oxidized form of Pt, which is present in the single-atom catalyst synthesized via atom trapping, meets the demands for thermal durability but does not exhibit high activity at low temperatures for CO oxidation (Fig. 1b). However, even this low activity Pt single-atom catalyst can serve as a perfect precursor to an exceptionally active CO oxidation catalyst^15^. This requires in situ treatment of the single-atom catalyst to form Pt clusters of suitable size, something that can be easily achieved using the engine control system in a modern automobile^16^. The atom trapping approach may therefore represent a possible scalable synthesis for thermally stable Pt, Pd, or Rh catalysts on ceria supports (Fig. 1b). Alternative approaches are being actively studied to achieve 100% dispersion, such as the solution-based atomic layer deposition used by Getsoian et al.^17^ at FORD to deposit Rh on TiO_2_, yielding thermally stable catalysts for emission control. By using nano-ceria stabilized on alumina, Jeong et al. at KAIST were able to achieve 100% dispersion of Pt, Pd, and Rh ensembles that were able to survive the severe 900 °C hydrothermal aging that is required for emission control catalysts^18^. The promise of single-atom catalysts is to lower the requirements for platinum group metals by utilizing these metals more efficiently. This was achieved recently by Khivantsev et al. at PNNL through the use of Rh at ultralow loadings (0.1 wt%) prepared via atom trapping for NO reduction^19^. In summary, recent research shows pathways for scalable synthesis of single-atom catalysts that might deliver catalysts meeting the thermal durability requirements of industry, while yielding reactivity improvements over conventional supported metal nanoparticle catalysts.

Over the past decade, single-atom catalysis has evolved from being an academic curiosity to one of the most widely researched methods for the synthesis of novel catalytic materials. New applications continue to emerge, including selective hydrogenation, selective oxidation, and complex organic synthesis for commodity chemicals^7^. Research on single-atom stability has an important side benefit—mobile single atoms constitute the dominant mechanism for catalyst sintering via Ostwald ripening. Stabilizing single atoms could therefore help improve the durability of all heterogeneous catalysts and might lead to new ways for catalyst regeneration, transforming metal nanoparticles back into single atoms. Challenges that remain in this field involve a better definition of the three-dimensional environment of single atoms, as well as the ability to tune the interaction with neighboring atoms and to control their oxidation state. STM is able to image the single atoms and the neighbors^20^, but cannot be applied to powder catalysts. Achieving similar resolution in industrial catalysts through a combination of XAS and AC-STEM along with DFT, and operando spectroscopies will help in the understanding of single-atom sites and their reaction mechanisms, leading to widespread applications in industrial practice.
